# The Potential Usefulness of Virtual Reality Systems for Athletes: A Short SWOT Analysis

**DOI:** 10.3389/fphys.2018.00128

**Published:** 2018-03-05

**Authors:** Peter Düking, Hans-Christer Holmberg, Billy Sperlich

**Affiliations:** ^1^Integrative & Experimental Exercise Science & Training, Institute for Sport Sciences, University of Würzburg, Würzburg, Germany; ^2^Swedish Winter Sports Research Centre, Mid Sweden University, Östersund, Sweden; ^3^School of Sport Sciences, UiT The Arctic University of Norway, Tromsø, Norway; ^4^School of Kinesiology, University of British Columbia, Vancouver, BC, Canada

**Keywords:** telemedicine, eHealth, mHealth, telerehabilitation, wearable, internet of sports

Virtual reality (VR) systems (Neumann et al., 2017), which are currently receiving considerable attention from athletes, create a two- or three-dimensional environment in the form of emulated pictures and/or video-recordings where in addition to being mentally present, the athlete even often feels like he/she is there physically as well. As she/he interacts with and/or reacts to this environment, movement is captured by sensors, allowing the system to provide feedback.

As with every newly evolving technology related to human movement and behavior, it is important to be aware of the strengths, weaknesses, opportunities and threats (SWOT) associated with the use of this particular type of technology. SWOT analyses are widely utilized for strategic planning of developmental processes (Pickton and Wright, 1998; Tao and Shi, 2016) and it is of great interest to consider whether VR systems should be adopted by athletes or not. Aspects more inherent to the employed technologies of VR systems, and aspects more related to the application of VR systems with athletes are considered as strength/weaknesses and opportunities/threats, respectively. Analogously, SWOT analysis concerning another emerging technology involving sensors of individual parameters (i.e., “implantables”) has been performed (Sperlich et al., 2017).

## Strengths

VR systems allow individualization of training (Kim et al., 2013) and can be applied even in everyday settings, such as when traveling, lying in bed or working. Moreover, (bio-)feedback (Düking et al., 2017) can be provided by continuous learning algorithms to athletes directly in real time (Kim et al., 2013) and/or even remotely to coaches (Neumann et al., 2017).

Inherent to the nature of VR is the potential to design and manipulate freely an almost infinite number of procedures for training athletes individually (Hoffmann et al., 2014). For example, manipulation of the visual environment (e.g., fog, light reflections, darkness, dust, rain, snow) allows many different conditions to be experienced. In addition, a large number of repetitions per training session can be achieved, which is likely to be beneficial in connection with sports where this is not possible in real life (e.g., ski jumping, downhill skiing, sky-jumps, and many more). In VR, an individual may compete against or train with any other athlete around the world (Capin et al., 1997; Neumann et al., 2017), regardless of their relative levels of performance, gender, ages and even if the other athlete is injured.

## Weaknesses

Realistic environments, which enhance the sense of immersion, are key to optimizing training and learning (Vignais et al., 2015).

The level of immersion depends on the feeling of “being present” in VR (place illusion) and the illusion of what is happening is real (plausibility illusion) (Slater, 2009). Consequently, the haptic, tactile, visual, and/or audio (bio-)feedback provided must be as realistic as possible and movements in the real world need to be synchronized with those in the virtual world (Vignais et al., 2015; otherwise, “seasickness” can be induced, Faisal, 2017). However, current VR systems cannot always achieve these goals (Katz et al., 2006).

Moreover, certain VR applications designed to capture the motion of athletes in real time require massive computational power, as well as a broad bandwidth for the transfer of data. Real video footage requires a relatively extensive database, whereas animated video footage may result in the “uncanny valley” effect, i.e., realistic graphical representations of characters that evoke unpleasant feelings (Vignais et al., 2015).

For a more realistic experience, the technology should be non-obtrusive, as small and light-weight as possible, allowing the athlete to execute movements without restriction or harming him/herself or others.

Finally, the costliness of setting up VR systems can limit their usage.

## Opportunities

VR systems enable athletes to learn remotely from any coach and at a time and place of their own choosing, improving a wide variety of skills such as decision-making and pacing strategies that optimize utilization of energy (Hoffmann et al., 2014; Murray et al., 2015; Romeas et al., 2015; Gokeler et al., 2016). Creative behavior, involving a wide variety of patterns of movement and tasks (Santos et al., 2016), can be stimulated by providing a plethora of appropriate exercises. Exercising in VR can lower the level of perceived exertion while simultaneously enhancing enjoyment (Mestre et al., 2011), which could increase the willingness to exercise, as well as performance while exercising.

Prior to competitions, VR systems can probably be employed to optimize warm-up procedures (Calatayud et al., 2010), for example, by enhancing motor imagery (Louis et al., 2008). Stress and certain dimensions of (competitive) anxiety could potentially be managed more efficiently with such systems (Parsons and Rizzo, 2008; Stinson and Bowman, 2014). With VR, athletes can train for competitions under the conditions predicted for the actual event, thereby achieving more realistic preparation (Swaren et al., 2012).

VR might also help injured athletes in two ways: First, it could aid the diagnosis of certain aspects of sport-related injuries (Teel and Slobounov, 2015). And secondly, recovery could be promoted by providing exercises designed to maintain mental alertness and readiness through simulation of real-life scenarios from a first-person perspective (Craig, 2014) and/or by helping athletes to maintain appropriate movements during rehabilitation (Fitzgerald et al., 2007; Gokeler et al., 2016).

From an employment perspective, specialized coaches will most likely have to be hired to implement and handle the more complicated VR systems of the future.

For researchers, VR provides exceptional opportunities for highly reliable field-testing of athletes (Gokeler et al., 2016), e.g., their perception-action-loops (Bideau et al., 2010; Craig, 2014). In the future, such diagnostic tests could also be applied routinely to young athletes, e.g., for earlier identification of talent.

## Threats

The transferability of skills, tactics, creative behavior and diagnostic procedures from the virtual to the real world remains to be established scientifically, although there is already evidence for the transferability of skills (Tirp et al., 2015). Some VR sensations (e.g., of g-forces, 3-D orientation) are currently not realistic, which could lead to unnatural patterns of movement, as well as under-/overuse and/or injury.

As with every novel technology, VR must first prove its value in order to convince rehabilitation specialists, athletes, coaches and others to adopt it (Katz et al., 2006; Akenhead and Nassis, 2015).

From an economic perspective, certain coaching jobs could be jeopardized by VR systems and, moreover, the cost of certain of these systems is still quite high.

Furthermore, VR systems may pose a threat to certain aspects of health, e.g., mental or visual (Spiegel, 2017). Proper hygiene must be given high priority, especially with respect to avoiding the spread of bacteria and/or viruses among team members (Davies et al., 2017). When exercising in VR, an athlete may be more prone to falling or collision with nearby objects, a risk which appears to be particularly great in connection with visual restriction due to a head-mounted display (Neumann et al., 2017). Another real risk associated with extensive use of VR systems in general is social isolation (Spiegel, 2017).

Finally, the personal data collected by VR systems must be protected from outside access and misuse (Spiegel, 2017).

## Summary

To summarize, VR systems show considerable promise for improving certain aspects of athletic performance, such as tactics or creative behavior, as well as in connection with rehabilitation, and research. Current technological limitations restrict sophisticated application of VR by athletes and transferability from the virtual to the real world and certain related health concerns require detailed further investigation.

Although SWOT analyses have potential limitations (e.g., by being too subjective; Pickton and Wright, 1998), we believe that this opinion article offers a valuable starting point for those who want to know more about the use of VR systems by athletes.

We have pointed out only the most prominent strengths, weaknesses, opportunities and threats associated with the use of VR systems in connection with sports (Table 1) and there are surely many more. It is noteworthy that most current research in this area focuses on aerobic sports and more emphasis on skill-based sports is needed (Neumann et al., 2017). Moreover, VR systems are still in their infancy and the substantial improvements and other alterations certain to come in the near future, as well as the applicability of VR systems to the athletic population must be monitored continuously and carefully.

**Table 1:**
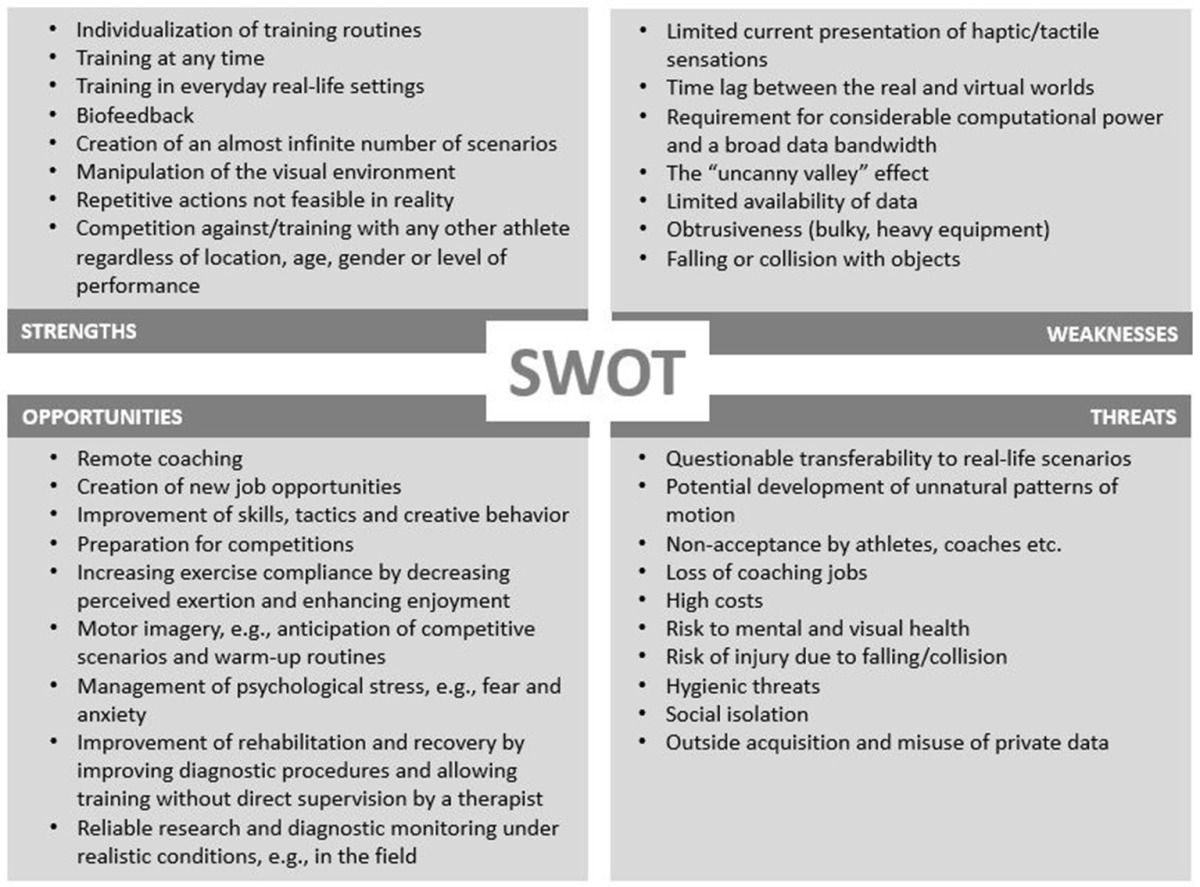
Strengths, weaknesses, opportunities, and threats associated with the use of VR systems by athletes.

## Author contributions

All authors listed have made a substantial, direct and intellectual contribution to the work, and approved it for publication.

### Conflict of interest statement

The authors declare that the research was conducted in the absence of any commercial or financial relationships that could be construed as a potential conflict of interest.
